# ATR Suppresses Endogenous DNA Damage and Allows Completion of Homologous Recombination Repair

**DOI:** 10.1371/journal.pone.0091222

**Published:** 2014-03-27

**Authors:** Adam D. Brown, Brian W. Sager, Aparna Gorthi, Sonal S. Tonapi, Eric J. Brown, Alexander J. R. Bishop

**Affiliations:** 1 Department of Cellular and Structural Biology, University of Texas Health Science Center at San Antonio, San Antonio, Texas, United States of America; 2 Greehey Children’s Cancer Research Institute, University of Texas Health Science Center at San Antonio, San Antonio, Texas, United States of America; 3 Abramson Family Cancer Research Institute, Department of Cancer Biology, University of Pennsylvania School of Medicine, Philadelphia, Pennsylvania, United States of America; 4 Cancer Therapy and Research Center, University of Texas Health Science Center, San Antonio, Texas, United States of America; CNR, Italy

## Abstract

DNA replication fork stalling or collapse that arises from endogenous damage poses a serious threat to genome stability, but cells invoke an intricate signaling cascade referred to as the DNA damage response (DDR) to prevent such damage. The gene product ataxia telangiectasia and Rad3-related (ATR) responds primarily to replication stress by regulating cell cycle checkpoint control, yet it’s role in DNA repair, particularly homologous recombination (HR), remains unclear. This is of particular interest since HR is one way in which replication restart can occur in the presence of a stalled or collapsed fork. Hypomorphic mutations in human *ATR* cause the rare autosomal-recessive disease Seckel syndrome, and complete loss of *Atr* in mice leads to embryonic lethality. We recently adapted the *in vivo* murine pink-eyed unstable (*p^un^*) assay for measuring HR frequency to be able to investigate the role of essential genes on HR using a conditional Cre/loxP system. Our system allows for the unique opportunity to test the effect of ATR loss on HR in somatic cells under physiological conditions. Using this system, we provide evidence that retinal pigment epithelium (RPE) cells lacking ATR have decreased density with abnormal morphology, a decreased frequency of HR and an increased level of chromosomal damage.

## Introduction

DNA damage is an unavoidable consequence of life resulting from both endogenous and exogenous sources. Dividing cells are particularly susceptible to DNA damage as many lesions can cause replication forks to stall and/or collapse. Without an appropriate response, such interruptions to DNA replication can lead to genome instability. To ensure that chromosomes are accurately and faithfully duplicated, cells have evolved an elaborate set of DDR mechanisms in which DNA replication slows allowing for the recruitment of DNA repair factors while also preventing potentially deleterious progression through cell cycle [1]. One such response to replication stress involves the protein kinase ATR. The ATR protein kinase is a member of the phosphoinositide 3 kinase (PIKK) family, and is the orthologue of *Saccharomyces cerevisiae* Mec1. Activation of ATR by replication-blocking DNA damage elicits a pleiotropic signal transduction pathway that includes numerous transducer and effector proteins [2].

Similar to many other DDR proteins, *ATR* is an essential gene and its absence leads to early embryonic lethality in mice prior to embryonic day 7.5 (E7.5) [3]. Furthermore, loss of ATR function via the disruption of the kinase domain also results in early embryonic lethality before E8.5 [4]. It is interesting to note that *bona fide* ATR heterozygous mice exhibited an increase incidence of tumors [3] and that ATR heterozygosity results in a decreased S-phase arrest [5]. Conclusions drawn from these earlier studies were that the lethality is likely due to a defect in checkpoint control in the presence of replication stress during a time of rapid cellular proliferation. Supporting this conclusion, subsequent studies have demonstrated that replication inhibition and DNA damage induce the formation of ATR foci [6], and ATR prevents the formation of DNA double strand breaks (DSBs) in response to stalled replication forks [7]–[9].

One of the mechanisms used by cells to resolve replication stress or a stalled replication fork, such that they can continue dividing, is HR repair [10]. This type of repair is considered a high fidelity process since it utilizes homologous sequences as an accurate template. This template is typically provided by the sister chromatid during S- or G2-phase before mitosis. Due to the correlation between replication stress-induced ATR activation and initiation of HR, a number of studies have investigated the role of ATR in regulating HR. Indirect evidence suggesting that ATR promotes HR include the findings that ATR phosphorylates a number of substrates known to directly affect HR (*e.g*. CHK1, BRCA1 and BLM) [6], [11]–[14]. Additionally, the PIKK inhibitor caffeine, which inhibits ATR kinase activity as well as other PIKKs such as ATM and DNA-PK, decreased site directed DSB-induced HR [15]. Further, reduced levels of ATR rendered cells sensitive to PARP1 inhibition [16], a phenomenon often associated with a HR defect. More direct studies addressing the role of ATR in HR have also been conducted. Following expression of a kinase dead ATR mutant protein, Wang *et al.*, found that HR frequency decreased following a restriction enzyme-mediated site-directed DSB, presumably in a dominant-negative fashion [17]. This would suggest that ATR promotes HR following DNA damage. In contrast, Chanoux *et al.* found that the conditional deletion of ATR in mouse embryonic fibroblasts resulted in an approximate two-fold increase in spontaneous-induced RAD51 foci (a surrogate marker of an early and essential step in HR), and that this result was further increased in the presence of the replication stress inducing agent aphidicolin [18]. However, it should be noted that though RAD51 protein is necessary in an early step of HR, its accumulation in nuclear foci does not necessarily indicate completion of the HR repair event.

In light of these discrepancies, we set out to investigate whether ATR deficiency affects the frequency of HR that is observed through normal development. For this study, we utilized the *p^un^ in vivo* mouse system for measuring HR. The basis of the *p^un^* system is a 70 kilobase tandem repeat of genomic material within the pink-eyed dilution (*p*; also known as *Oca2*) gene, rendering it functionless [19]–[21]. The *p* gene is involved in pigmentation, so the *p^un^* mouse is identified phenotypically by the appearance of a light grey coat and pink eyes (due to a colorless RPE cell layer). Within the eye, the RPE is normally a pigmented cell type, and restoration of this pigmentation in otherwise transparent cells is the basis of our assay. To produce a functional *p* gene in RPE cells carrying homozygous *p^un^* mutation, a deletion-mediated recombination event must occur between the duplicated region, deleting one copy of the 70 kb region and establishing the correct intron/exon format of the wild-type gene. Based upon studies using an analogous system in yeast [22], as well as genetic and exposure studies with the *p^un^* assay [23]–[25] conducted by others and our laboratory, it seems that these recombination events could occur via single strand annealing (SSA), unequal crossing over or gene conversion either between sister chromatids or homologues or via a template switch event during DNA replication. To assess the role of essential genes like *Atr* on HR, we recently modified the *p^un^* assay, to include the tissue-specific *Cre/loxP* system [24] and now extend this system to investigate the *in vivo* conditional loss of ATR on HR. Our findings suggest a role for ATR in promoting HR and that its absence results in chromosomal instability and cellular abnormalities.

## Materials and Methods

### Mouse lines

C57BL/6J pink-eyed unstable (*p^un/un^*) mice were obtained from Jackson Laboratory. The *p^un^* mutation is a recessive mutation, so genotyping homozygosity of this allele is through the appearance of a dilute (*i.e.* grey) fur coat and pink eyes. *Atr^+/neo^* [3] and *Atr^flox/flox^* [9] were obtained from E.J. Brown on a C57BL6 background and backcrossed two times to *p^un/un^* mice. Cre expressing (*Trp1-Cre^tg/tg^ p^un/un^*) mice and Cre activity reporter based on expression of nuclear localized beta-galactosidase (β-gal) (*RC::PFwe^ki/ki^ p^un/un^*) [26] were used to establish two cohorts of mice in a manner similar to the study by Brown *et al*. [24]; 1) *Atr* constitutive (*Atr^+/−^ Trp1-Cre^tg/tg^ p^un/un^*) and 2) *Atr* floxed (*Atr^flox/flox^ RC::PFwe^ki/ki^ p^un/un^*). Animals from each cohort were crossed to generate *Atr* conditional heterozygous (*Atr^cond/+^ Trp1-Cre^tg/o^ RC::PFwe^ki/+^ p^un/un^*) and *Atr* conditional null (*Atr^cond/−^ Trp1-Cre^tg/o^ RC::PFwe^ki/+^ p^un/un^*). The study was approved by the University of Texas Health Science Center at San Antonio Institutional Animal Care and Use Committee (IACUC) policy as outlined in our protocol number 07005-34-02-A,B1,C. The facility is operated in compliance with the Public Law 89–544 (Animal Welfare Act) and its amendments, Public Health Services Policy on Humane Care and Use of Laboratory Animals (PHS Policy) using the Guide for the Care and Use of Laboratory Animals (Guide) as the basis of operation. Periodic inspections are conducted by the United States Department of Agriculture (USDA). The University is accredited by the Association for Assessment and Accreditation of Laboratory Animal Care, International (AAALAC). Mice were euthanized using procedures recommended by the Panel of Euthanasia of the American Veterinary Medical Association, namely CO2 asphyxiation with CO2 delivered from a compressed gas cylinder by inhalation to effect in a chamber that has not been recharged.

### Retinal pigment epithelium dissection and whole mount staining

Eyes were removed, rinsed in phosphate buffered saline (PBS), fixed in 4% paraformaldehyde (PFA), rinsed again in PBS and stained for β-galactosidase activity as previously described [24]. Stained eyes were dissected and RPE whole mounts were mounted according to Claybon and Bishop [27]. For those RPE whole mounts that were stained with phalloidin, Alexa Fluor 546 Phalloidin (Molecular Probes, Life Technologies) was used according to manufacture instructions. In brief, PFA fixed RPE whole mounts were rinsed in PBS and then blocked in 1% bovine serum albumin for twenty minutes at room temperature. Phalloidin stock reagent was diluted 1:40 in blocking solution and incubated in the dark for an additional twenty minutes. Samples were washed three times in PBS, mounted onto glass microscope slides and imaged.

### Visualization and analysis of whole mounts

All RPE whole mounts were visualized using a Zeiss Lumar V.12 stereomicroscope and Zeiss Axiovision 4.6 software. Measurements of RPE length were performed using Adobe Photoshop, and the reported RPE petal length was defined as the distance from the optic nerve to the distal edge of the RPE. Relative RPE petal length was calculated by dividing the petal length for each sample per genotype by the average petal length of the *p^un/un^* samples. To determine cell density, a 200 μm^2^ area was drawn using Adobe Photoshop at the position that is approximately 0.6 of the petal length from the optic nerve head to the edge of the RPE and the total number of cells located completely inside this area were counted. If any portion of a cell was found to be outside of this area, then it was excluded from counting. The same 200 μm^2^ area used for cell density was also used for quantifying micronuclei (see results for description) with the exception that any cell (*i.e.* touching or entirely inside the area) was used. Therefore, we have used this region as a representation of the entire RPE and reported the data as the percentage of cells with micronuclei. The observation of cellular morphology and quantifications of cell density and micronuclei were done using the phalloidin-stained images in order to accurately identify cell boundaries. Using these fluorescent-based images meant that β-galactosidase positive staining nuclear material (*i.e.* blue nuclei and blue micronuclei) appears black and not blue.

RPE HR reversion events (*i.e.* eye spots) were scored by the phenotypic appearance of a cell with their cytoplasm packed with brown melanosomes. Unless otherwise noted, the number of pigmented cells or groups of pigmented cells are counted for the entire RPE whole mount by using a stereomicroscope with criteria for what constitutes a single eye spot (single HR event) set forth by Bishop *et al.* [28]. To define Cre activity in our system we used a nuclear localized β-galactosidase Cre reporter system [24], [26]. The nuclear localization of this enzymatic reporter is paramount to our system because it allows for the detection of both Cre activity (nuclear blue stain) and HR (cytoplasmic brown pigmentation) in the same cell. For each eye spot counted, the presence of a β-galactosidase positive stained nucleus was also recorded. Therefore, the frequency of eye spots per RPE with or without β-galactosidase staining was used to calculate the frequency of HR (*i.e.* number of eye spots per RPE). Additionally, the overall percentage of β-galactosidase staining for each RPE whole mount was visually assessed and assigned a percentage.

### I-*Sce*I DR-GFP HR assay

The U2OS cell line containing a stably integrated copy of the direct repeat-green fluorescent protein (DR-GFP) construct was kindly provided by Dr. Maria Jasin, and the frequency of HR was measured according to previous publications [29], [30]. Briefly, Liopfectamine RNAiMAX (Invitrogen) was used to transfect 75 picomoles of either scramble control siRNA (Santa Cruz sc-37007) or ATR siRNA (Santa Cruz sc-29763) into 1×10^5^ DR-GFP U2OS cells using the reverse transfection method according to the manufacturer’s protocol. Cells were then washed 24 hours later and transfected with I-*Sce*I expression vector, empty vector or a GFP expression vector using Lipofectamine 2000 (Invitrogen) as an internal control for transfection efficiency. After 72 hours, cells were trypsinized and HR frequency was quantified as percentage GFP^+^ cells via flow cytometry. The experiment was run in triplicate for each condition. Knockdown efficiency was measured by western blot using standard methods (1∶1000 dilution of ATR antibody, Abcam ab2905). All statistical analyses were performed using GraphPad Prism (GraphPad Software, Inc.).

## Results

### Size reduction in ATR conditional null eyes

**Figure 1:**
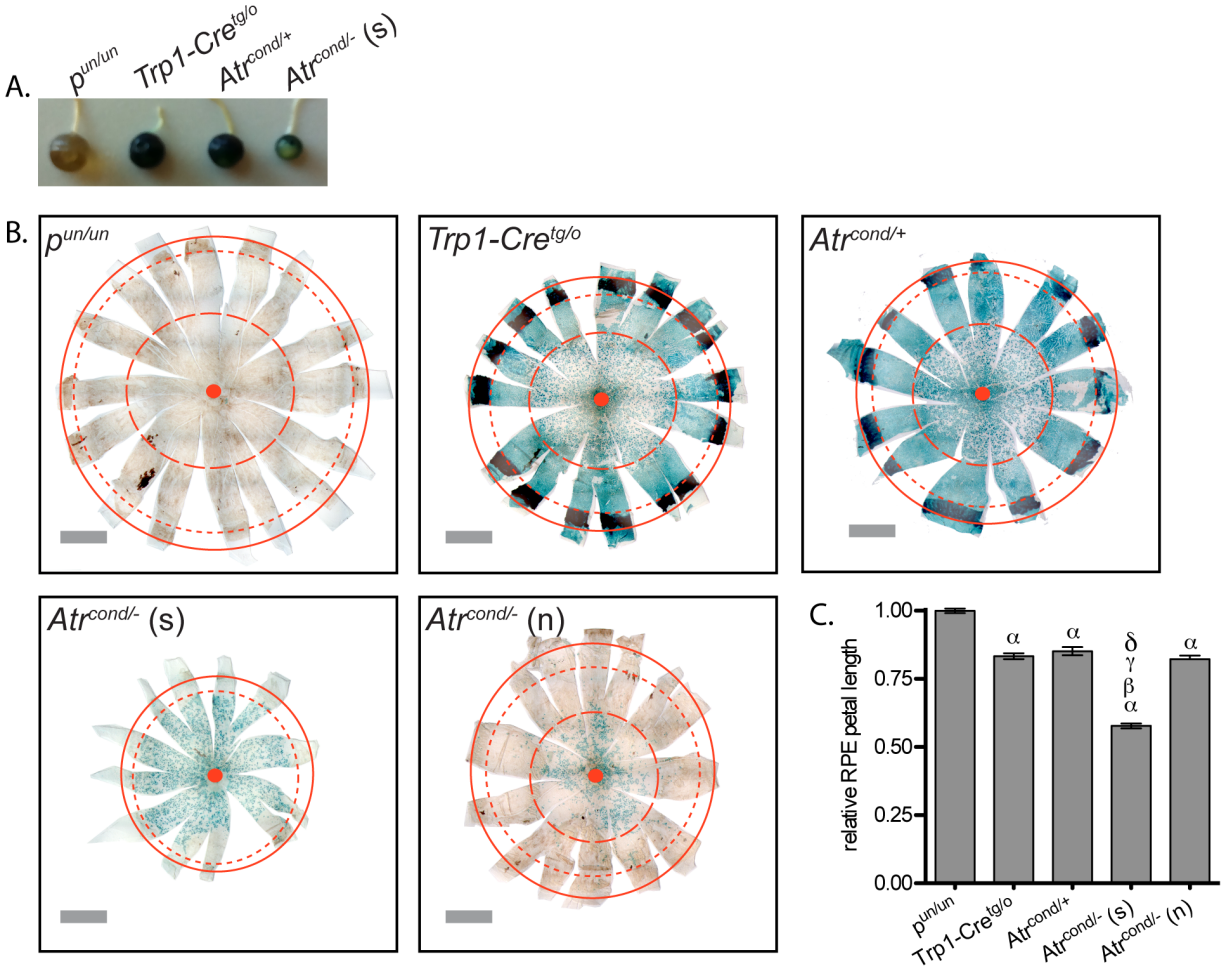
Conditional deletion of *Atr* leads to a reduction in the size of mouse eyes. Extracted eyes (**A**) and dissected RPE whole mounts (**B**) from 30 day old mice following nuclear localized β-gal activity staining. Conditional deletion of *Atr* often resulted in the significant reduction of eye size (**A** and **B**). The conditional loss of ATR significantly reduced RPE petal length as defined by the distance from the optic nerve to the distal edge of the RPE (**C**). i *p^un/un^* (n = 7); ii *Trp1-Cre^tg/o^* (n = 6); iii *Atr^cond/+^* (n = 6); iv (s) *Atr^cond/−^* small (n = 10); iv (n) *Atr^cond/−^* normal (n = 6) (**A**, **B** and **C**). Solid red lines indicate the distal edge of the RPE, small dashed red lines indicate the proximal edge of the RPE, large red dashed lines denote the region that is 0.6 of the petal length (petal length equals the distance between the optic nerve and the proximal edge of the RPE) and solid red circles indicate the optic nerve. Scale bar: 1 mM (**B**). For (**C**) α: comparison to *p^un/un^* (*P<0.0001*); β: comparison to *Trp1-Cre^tg/o^* (*P<0.0001*); γ: comparison to *Atr^cond/+^* (*P<0.0001*) and δ: comparison to *Atr^cond/−^* normal (*P<0.0001*); error bars indicate S.E.M.

It has been demonstrated that ATR is essential for embryonic development [3], [4], as well as being necessary for maintenance and homeostasis of tissues in adult mice using a ubiquitously expressing inducible Cre system [31]. We recently developed a conditional system to assess the role of essential genes on HR frequency by excising the gene of interest only in the RPE [24]. Expression of the Cre transgene is driven by the tyrosinase related protein 1 (Trp1) promoter, whose expression is restricted to the RPE during mouse embryonic development [32]. Therefore, we wanted to assess the effect of ATR loss on RPE development and HR frequency. We initially observed that the well-stained *Atr* conditional null eyes were markedly reduced in size (Fig. 1a) and further observation of RPE whole mounts confirmed this finding (Fig. 1b). We quantified this reduction in size by determining the change of petal length relative to *p^un/un^* samples (Fig. 1c). In doing so, we observed an approximate 45% reduction in RPE petal length of *Atr* conditional nulls compared to *p^un/un^* samples (*P<*0.0001, ANOVA with Tukey’s multiple comparison test). The *Trp1-Cre^tg/o^* and *Atr* conditional heterozygous eyes were also smaller than *p^un/un^* samples (*P<*0.0001, ANOVA with Tukey’s multiple comparison test), yet both were larger than the *Atr* conditional nulls. Therefore, the reduction in RPE petal length that can be attributed solely to loss of ATR is approximately 30%. In addition to the small ATR null eyes, we also observed a number of eyes of this same genotype that are apparently normal in size (Fig. 1b). Interestingly though, the proportion of β-galactosidase positive cells (*i.e*. denoting Cre activity) is substantially decreased in these RPE (Fig. 1b). Petal length of these RPE is similar to the *Trp1-Cre^tg/o^* and *Atr* conditional heterozygous eyes and larger than the small *Atr* conditional null samples (*P<*0.0001, ANOVA with Tukey’s multiple comparison test) (Fig. 1c).

### Loss of ATR results in abnormal RPE cellular morphology and increase in chromosomal instability

**Figure 2:**
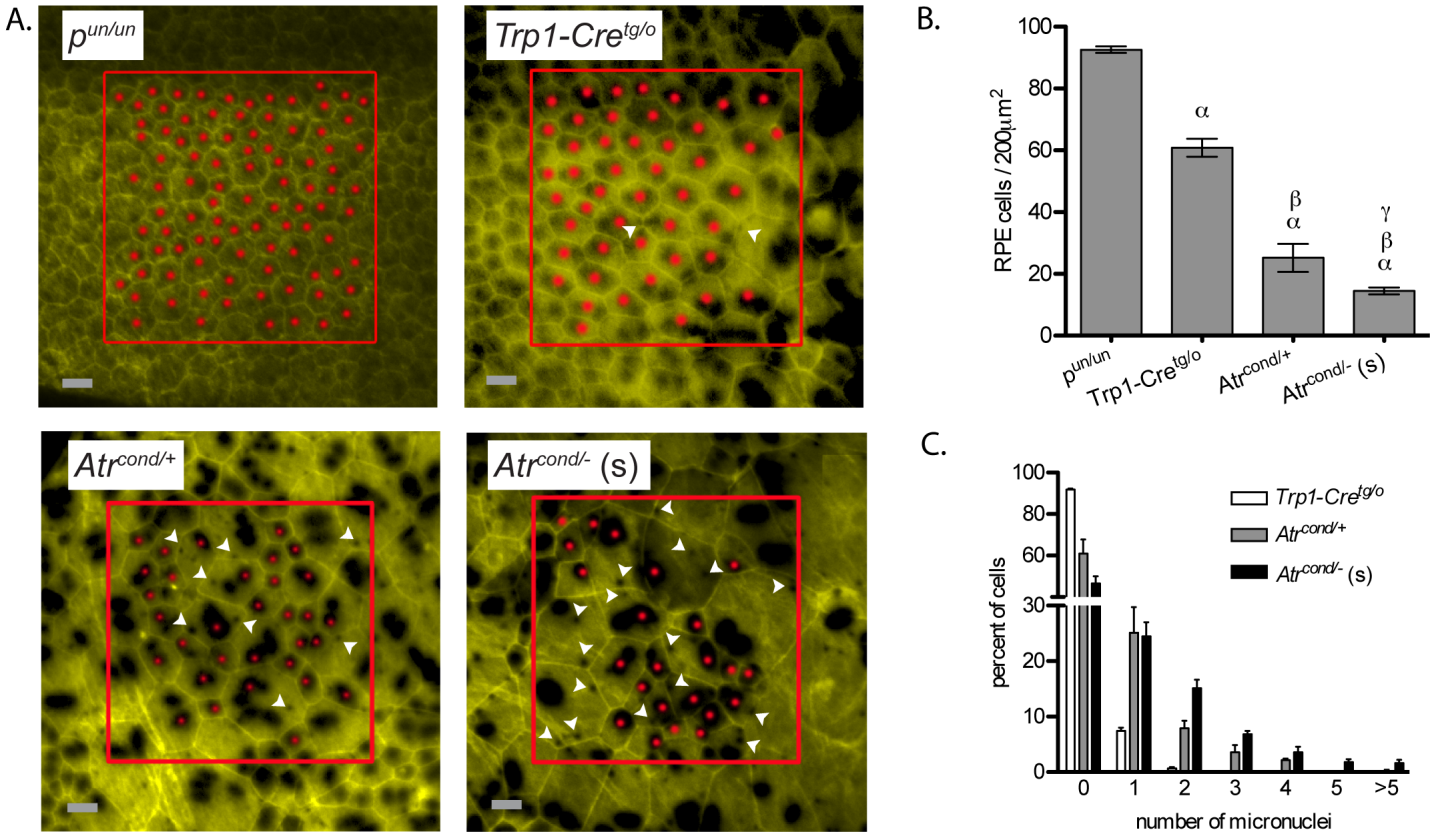
Morphological abnormalities of the RPE monolayer and increased chromosomal damage following the conditional deletion of *Atr*. Loss of either a single copy of *Atr* or both copies of *Atr* lead to morphological abnormalities of the RPE (A), a significant decrease in cell density (B) and a significant increase in micronuclei formation (C). i *p^un/un^* (n = 5); ii *Trp1-Cre^tg/o^* (n = 7); iii *Atr^cond/+^* (n = 9); and iv (s) *Atr^cond/−^* small (n = 23); (A, B and C). RPE whole mounts were stained for nuclear localized β-gal activity (black spots) and phalloidin (yellow) to identify nuclear material and cell boundaries, respectively. Red boxes indicate the 200 μm^2^ region used for cell counting, solid red circles and white arrowheads mark individual cells and micronuclei, respectively (A). Error bars indicate S.E.M. (B and C). Scale bar: 25 μm (A). For (B) α: comparison to *p^un/un^* (*P<0.001*); β: comparison to *Trp1-Cre^tg/o^* (*P<0.001*) and γ: comparison to *Atr^cond/+^* (*P<0.01*).

The cells of *p^un/un^* RPE exhibited a uniform cobble stone morphology, yet we observed varying degrees of differences from this norm in cells of *Trp1-Cre^tg/o^*, *Atr* conditional heterozygous and *Atr* conditional null RPEs, and these differences were most apparent in the *Atr* conditional null in which the cells were markedly increased in size with abnormal morphology (Fig. 2a). We therefore quantified cell density for each genotype and found that the loss of ATR was associated with a decrease in cell density and increase in cell size (Fig. 2b).

In order to compare across the genotypes, we used a 200 μm^2^ area at a position that is approximately 0.6 of the petal length from the optic nerve head to the edge of the RPE (Fig. 1b). This region was chosen because it is approximately the point in which the cells of the *Atr* conditional heterozygous RPEs regained a more wild-type (*e.g.* cobblestone-like) morphology. It is interesting to note that previous work using the *p^un^* system found that this region corresponded to the onset of the last third of embryonic RPE development [33], and this stage of embryonic development is when *Trp-1 Cre* expression within the RPE was previously noted to begin decreasing [32]. Furthermore, the distance that is 0.6 of the length of the *Atr* conditional heterozygous or *Trp1-Cre^tg/o^* RPE is approximately the same as the total petal length of the small *Atr* conditional null RPE (optic nerve head to the proximal edge) and where the cells of the more normal-sized *Atr* conditional null RPE no longer stained positive for Cre activity (*i.e*. lack of blue β-galactosidase stain) (Fig. 1b), suggesting that Cre is most likely not active from this point onward. The greatest loss in cell density was in RPE of the small *Atr* conditional null samples when compared to either *p^un/un^* (*P<*0.001, ANOVA with Tukey’s multiple comparison test), *Trp1-Cre^tg/o^* ( *P<*0.001, ANOVA with Tukey’s multiple comparison test) or *Atr* conditional heterozygous (*P<*0.01, ANOVA with Tukey’s multiple comparison test) (Fig. 2b). Cell density appears to be ATR dose dependent as *Atr* conditional heterozygous RPE were significantly decreased from *Trp1-Cre^tg/o^* (*P<*0.001, ANOVA with Tukey’s multiple comparison test). Normal sized *Atr* conditional null RPE were excluded from this analysis due to the lack of Cre activity in this region, as this indicates that these cells are actually *Atr* heterozygous (*i.e*. *Atr^flox/−^*).

The reporter employed in our system is a Cre-mediated nuclear localized β-galactosidase [26]. Unlike our previous study and unpublished data in which blue stain was restricted to the nuclei of these now fully differentiated post-mitotic RPE cells, we observed small cytoplasmic bodies of blue stain in addition to the more usual large blue nuclei (Fig. 2a, white arrow heads) in the ATR conditionally deleted RPE cells. A previous report using an ATR hypomorphic Seckel syndrome cell line described an approximate two-fold increase of spontaneous micronuclei compared to wild-type and this difference was further augmented following treatment with the replication stress-inducing drug hydroxyurea [34]. Based on the location and size of the small blue staining bodies, as well as the study by Alderton *et al*., we believe that it is reasonable to assume that these small blue stained bodies are micronuclei. We next quantified these blue bodies in the RPE of our conditional samples and found an inverse correlation between the amount of ATR and levels of micronuclei (*P<*0.0001, Chi-square test) (Fig. 2c). Interestingly, much like the observed change in cell density, *Atr* conditional heterozygosity resulted in a micronuclei haploinsufficiency phenotype and may reflect the decreased S-phase checkpoint previously observed with *ATR* heterozygous cells [5].

### ATR promotes homologous recombination in mouse RPE cells

**Figure 3:**
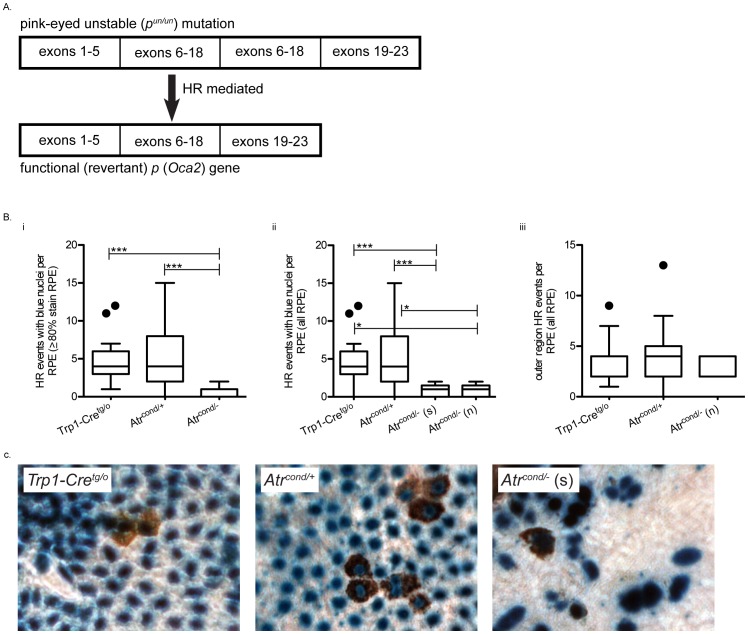
Spontaneous homologous recombination repair is decreased in the absence of ATR *in vivo*. Schematic representation of the *p^un^* mutation (tandem duplication of exons 6–18) in which a HR-mediated event facilitates the deletion of one copy of the repeat resulting in the reversion of the mutant allele to a functional *p* gene. Reversion (HR) events are scored phenotypically by counting the numbers of single or groups of cells in the RPE with brown pigmentation in their cytoplasm. (**A**). Loss of one copy of *Atr* does not affect HR frequency (*i.e.* the number of *p^un^* reversion events per RPE) (**Bi** and **ii**), whereas complete loss of ATR resulted in the significant decrease of HR frequency (**Bi** and **ii**). Within the outer clear region of *Atr^cond/−^* normal eyes (*i.e.* no β-gal activity suggesting the presence of a single copy of *Atr*), the HR frequency was not different from *Trp1-Cre^tg/o^* and *Atr^cond/+^* in similar regions (**Biii**). Representative eye spot images from different genotypes in B. Pigmentation can be observed in the cytoplasm following a *p^un^* reversion event in the RPE. Blue nuclei indicate nuclear-localized Cre activity (**C**). For (**B** and **C**) (s) *Atr^cond/−^* small and (n) *Atr^cond/−^* normal eyes; *P<0.05 and ***P<0.001.

We next assessed the frequency of *p^un^* reversion events (Fig. 3a) in our samples by quantifying the presence of pigmented eye spots per RPE. We previously quantified HR frequency using three different criteria: 1) all eye spots regardless of nuclei color; 2) eye spots with blue nuclei (*i.e.* β-galactosidase positive stained nuclei to denote Cre activity occurred) and 3) eye spots with blue nuclei from RPE with an overall blue stain of ≥80% [24]. Using the most stringent criteria, we found that the frequency of HR is significantly decreased in the *Atr* conditional null RPEs compared to *Trp1-Cre^tg/o^* and *Atr* conditional heterozygous (*P<*0.001, Kruskal-Wallis with Dunn’s multiple comparison test) (Fig. 3bi and 3c). Whereas *Atr* haploinsufficiency impacted cell density and occurrence of micronuclei, the frequency of HR in *Atr* conditional heterozygotes was not different from *Trp1-Cre^tg/o^* (Fig. 3b). To account for the different sized conditional null samples we separated out small and normal sized RPE and reanalyzed them. In order to do this, we had to reduce the stringency criteria to include all eye spots regardless of nuclei color. Though this reduced stringency likely results in the inclusion of events that occurred without deletion of Atr (thus inflating the HR frequency), it was necessary to allow inclusion of additional samples because the majority of the normal sized null samples had limited blue staining. The frequency of HR for both the small (*P<*0.001, Kruskal-Wallis with Dunn’s multiple comparison test) and normal (*P<*0.05, Kruskal-Wallis with Dunn’s multiple comparison test) sized *Atr* conditional null samples were significantly decreased compared to *Trp1-Cre^tg/o^* and *Atr* conditional heterozygous (*P<*0.0001, Kruskal-Wallis with Dunn’s multiple comparison test) (Fig. 3bii), but not different from each other. In order for this analysis to be valid, we assume that all HR eye spots lacking blue nuclei were actually conditionally null rather than remaining heterozygous. Our previous work though, found that the amount of blue stain strongly correlated with excision of our gene of interest [24]. Therefore, we next asked if in the approximate outer one third of the normal sized null eyes whether the HR frequency is similar to that of *Trp1-Cre^tg/o^* and *Atr* conditional heterozygous samples (Fig. 1b). This region was found to be devoid of blue stain suggesting that the floxed allele of *Atr* in these cells was not excised (*i.e.* the cells in this region are heterozygous for *Atr*). As expected from our previous analysis, the HR frequency in this region was similar to that of the *Trp1-Cre^tg/o^* and *Atr* conditional heterozygous groups (Fig. 3biii).

### ATR promotes homologous recombination in a human tissue culture assay

**Figure 4:**
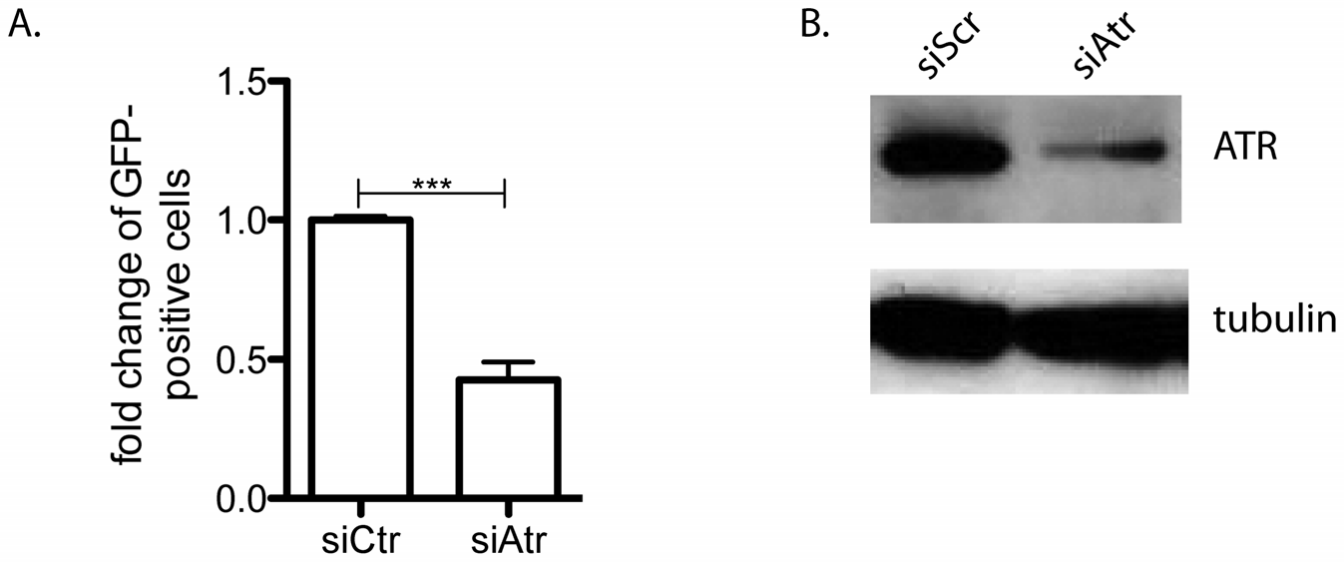
ATR promotes homologous recombination *in vitro*. *I-SceI*-induced HR-mediated repair was significantly decreased in the DR-GFP U2OS cell line with reduced ATR expression (**A**). For (**A**) ***P<0.001 and n = 3. (B) A representative image of ATR expression knockdown using siRNA in the DR-GFP U2OS cells (siCtr is scramble).

Finally, to corroborate our *in vivo* data, we used the well-established DR-GFP *in vitro* HR reporter assay [29], [30] to examine HR in cells lacking ATR. ATR expression was decreased using siRNA targeted to human *ATR* in the U2OS cell line that stably expresses the DR-GFP construct [35]. The frequency of I-*SceI*-induced HR was two-fold lower in cells with decreased ATR expression compared to scramble control siRNA cells (*P*  =  0.0009, unpaired t-test) (Figs. 4a and 4b), matching what was previously reported with either caffeine exposure [15] or expression of a presumably dominant negative ATR kinase dead mutant [17]. This result also suggests that ATR may have a more general role in controlling homologous recombination in response to a double strand break, having already been shown to be involved in some of the latter parts of break processing [36].

## Discussion

Genomic instability is known to be a major factor in cancer development, thus understanding the systems that influence genomic stability in the normal somatic tissue is key to understanding cancer predisposition. Homologous recombination is a key DNA repair pathway that is usually considered to have high fidelity. However, either too much or too little HR can be deleterious, resulting in genomic alterations that can promote cancer development. To examine HR through normal somatic development we use the *p^un^* assay system, often observing changes that are too infrequent to be observed by tissue culture systems and without confounding issues of multiple altered genetic backgrounds as are often present in established tissue culture systems. We have used this strategy successfully for a number of genetic models with differing results; ATM, p53, GADD45a, BLM, and PARP1 suppress *p^un^* events, while BRCA1 and BRCA2 are necessary for a subset of *p^un^* events [24], [25], [33], [37], [38]. Although the initiating lesion in the *p^un^* assay is unknown, data from our laboratory and others predict that spontaneous HR events can be initiated in response to DNA replication stress [22], [24], [33]. Additionally, Saleh-Gohari *et al.* demonstrated that endogenous damage (*e.g.* DNA single strand breaks) is most similar to damage-induced collapsed replication forks, and that these lesions are most likely substrates for spontaneous HR events [39].

**Figure 5:**
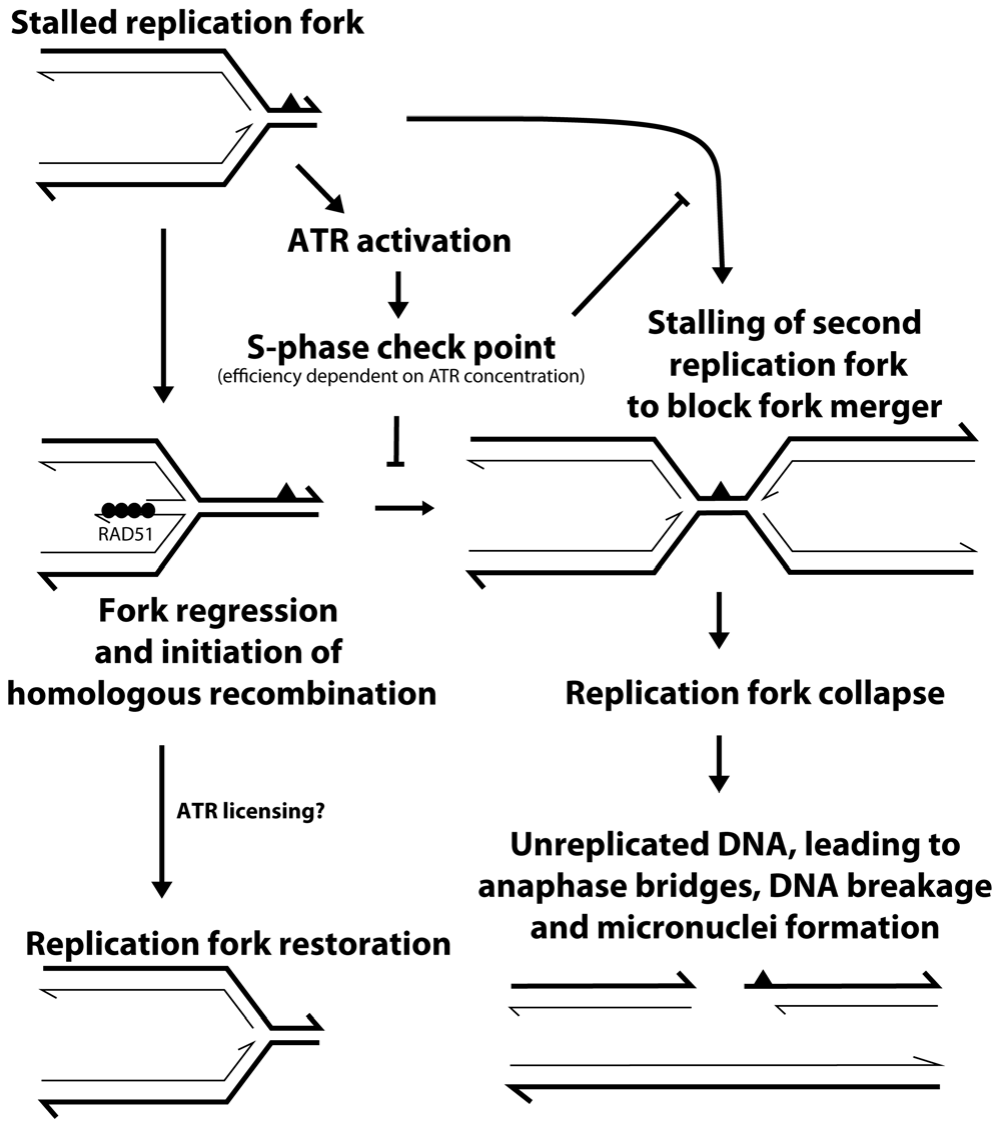
Model for the relationship between ATR, homologous recombination and chromosomal stability. In the presence of replication stress from endogenous lesions, ATR is activated initiating an S-phase arrest to block aberrant merger of another replication fork at the same lesion. Some stalled replication forks will be substrates for HR (depicted here is the formation of a chicken foot structure that acts as a RAD51 substrate). If HR proceeds as normal, then the replication fork will be restored. However, we propose that this progression is dependent upon sufficient time to complete the HR reaction and possibly a more direct licensing of a later step in HR by ATR kinase activity (CHK1, BRCA1 and BLM, for example – not shown). If HR does not restore the stalled fork, then it may collapse, potentially leading to chromosomal breaks and the production of micronuclei. Thick lines are parental strand DNA, thin lines are daughter strand DNA, half arrowheads represent 3′ ends, the solid black triangle represents a DNA lesion and solid black circles represent RAD51 protein.

The primary objective of this study was to assess the effect of ATR loss on spontaneous HR frequency. In order to bypass the embryonic lethality associated with complete loss of ATR [3] or *Atr* kinase dead [4] mouse models, we utilized the recently developed *in vivo* conditional mouse *p^un^* assay [24]. In the current study, we found that conditional loss of ATR led to a 2.5-fold reduction in the frequency of spontaneous HR, suggesting that ATR promotes this type of repair (Fig. 3bi). In response to site-directed DSBs, Wang *et al*. also showed a 2-fold reduction in ATR kinase dead cells [17], and we also observed a similar reduction of HR following ATR knockdown using a similar *in vitro* assay (Fig. 4). ATR is believed to be the primary signaling kinase responding to replication stress in order to prevent deleterious lesions (*e.g*. replication fork collapse or DSBs) that might be caused by replication fork stalling. Previous studies have found that ATR deficient cells have increased levels of the DSB marker H2AX following exposure to agents that induce replication stress [9], [18]. Therefore, we suggest that ATR reduces the incidence of DSBs that can occur as a result of replication fork collapse by promoting HR at a stalled replication fork (Fig. 5). In apparent contrast to this model, an increase in both spontaneous and replication damage-induced RAD51 foci in the absence of ATR has been reported [9], [18]. A potential explanation for this discrepancy is that accumulation of RAD51 foci measures HR initiation [40], which may not be inhibited by the absence of ATR, while the study by Wang *et al.* and the work presented here measured the completion of an HR event. Therefore, it is plausible to hypothesize that ATR function is not required for the initiation of HR, but rather a later step that results in the removal of RAD51, thus allowing for the completion of HR. Alternatively, but not necessarily mutually exclusively, the absence of ATR and a lack of S-phase arrest may not allow sufficient time for the completion of HR before a merging replication fork will stall at the same lesion. Such an event would result in replication fork collapse, DNA breakage and thus micronuclei formation. Such a “replication catastrophe” with ATR deficiency was recently described [41] and shown to result from depletion of RPA. RPA normally binds single stranded DNA during DNA replication and is known to interact with homologous recombination proteins to facilitate this DNA repair reaction [42].

Of note with the current study, in comparison to prior studies using the *p^un^* assay, the conditional loss of ATR is the only genetic alteration that has resulted in a size reduction of the eye and RPE. Furthermore, fragmented nuclei as identified by nuclear localized β-galactosidase staining in the conditional *p^un^* system, have only been observed in the partial and complete loss of ATR. Both of these unique observations attest to the hypothesis that ATR is essential for mitigating endogenous-derived genomic instability, most likely resulting from replication stress.

Homologous recombination is an essential process as evident by the number of genes involved in HR that are essential for mouse embryonic development [43]. This is in part due to increased levels of DNA damage and subsequent genomic instability. As mentioned above, cells lacking ATR have increased levels of DNA damage, and we also observed the product of an increased level of DNA damage in ATR deficient RPE, the accumulation of micronuclei (Fig. 2a and c). Our data therefore further supports a model in which a reduction in HR from loss of ATR leads to an increase in DNA damage and chromosomal instability (Fig. 5). This is also evident from the loss of RPE cellular morphology. It is noteworthy to mention that a recent study investigated the affect of TrpCre-1 on RPE biology. In this paper, the authors showed that Cre alone caused a decrease in cell density with an increase in abnormal cell morphology [44]. We also saw this phenomenon in our *Trp1-Cre^tg/o^* RPEs, but loss of ATR greatly augmented these effects. Perhaps this is due to the cell’s inability to properly respond to DNA damage induced from illegitimate Cre activity [45]. However, the ability of RPE cells to persist through to adulthood with this type of DNA damage having occurred during embryonic development, and the ensuing chromosomal instability, micronuclei and multiple nuclei, suggests something more. RPE cells frequently undergo azygotic mitosis towards the end of the development of the tissue, resulting in many RPE cells with two nuclei (but not more). This suggests that these cells may be able to retain viability when mitotic division is unsuccessful or smaller micronuclei are formed, despite being a primary tissue. This may explain the presence of multi nucleated and enlarged RPE cells when ATR is deficient. In a recent publication, Eykelenboom *et al* described a similar phenomenon following loss of ATR in the DT40 tissue culture system, however their cells died following the inappropriate progression through G2-M without completion of replication [46]. However, their results substantiate the requirement of ATR in preventing cell division in the absence of complete DNA replication and that without this checkpoint the production of micronuclei will ensue. Of note, the *p^un^* assay relies on the specific deletion of chromosomal DNA, generally considered a deleterious event due to the potential loss of genetic material. Our results would suggest that such genetic loss is preferential to the more catastrophic consequence of continuing through cell cycle without completing DNA replication.

Although *Atr* conditional heterozygous RPE did not display a HR haploinsufficiency phenotype, we did observe that *Atr* heterozygosity impacted both accumulation of micronuclei and cell density (Fig. 2). It is interesting to compare this observation with Seckel syndrome, the human disease associated with *ATR* mutation. Seckel syndrome results from the hypomorphic effect of inheriting a single *ATR* mutation, which consequently reduces ATR function and is characterized by severe microcephaly and proportional primordial dwarfism and skeletal abnormalities [47]. One cellular phenotype of Seckel syndrome is increased spontaneous and replication stress-induced micronuclei [34]. This is in agreement with the present study where we observe that both *Atr* conditional null, as well as *Atr* heterozygous cells has increased micronuclei frequency (Fig. 2c). Of interest, we have not observed these micronuclei when we used the same system to conditionally delete BRCA1 or BRCA2, similarly impairing HR (*data not shown*). If the absence of ATR results in increased replication fork collapse, DSBs and an inability to utilize HR to repair these lesions, then one would expect chromosomal fragmentation and thus micronuclei. However, we also noted a significant increase in micronuclei when only one copy of ATR was present, suggesting increased spontaneous damage and ensuing chromosomal instability. This observation fits with the known decreased S-phase arrest associated with *ATR* heterozygosity [5].

A recent report by Ruzankina *et al.* also conditionally deleted ATR in adult mouse tissues. The authors concluded that loss of ATR caused the premature appearance of age-related phenotypes and increased the deterioration of tissue homeostasis [31]. Similar to that study, we also observed a mosaicism of *Atr* deletion in our RPE, particularly in the normal sized *Atr* conditional null RPE (Fig. 1b). This is most pronounced in the more distal region of RPE where proliferation is greatest (many tightly packed cells produced in a short period at the end of RPE development), and suggests that a number of cells escaped Cre activity thereby remaining heterozygous and were capable of proper organ development. Supporting this interpretation of our observation is that the petal length (Fig. 1c) and HR frequency of the outer region without staining (therefore lacking Cre activity) (Fig. 3biii) of the normal sized *Atr* conditional null eyes are not different from *Atr* conditional heterozygous and *Trp1-Cre^tg/o^* RPE. These observations also belie the inherent issues of trying to measure HR in the context of modulating an essential gene such as *Atr*. In any such study it is necessary to control for the competition between the cells impaired in progression through cell division and proliferation because of the loss of an essential gene with cells that have a growth advantage due to retaining a functional copy of the essential gene. As such, our result that demonstrates the requirement of ATR in HR is probably a conservative measurement.

This study is another example of the ability of the conditional *p^un^* assay in measuring the effect of essential genes on HR frequency. More importantly, we have provided direct evidence that ATR is necessary for completion of HR events during somatic development in response to the normal levels of endogenous replication stress that occurs. The reduction of HR in the absence of ATR correlates with increased DNA damage and abnormal cell morphology.
